# Video Microscope Robotic Arm-Assisted, Neuronavigation-guided Glioma Resection and Regional Sampling

**DOI:** 10.7759/cureus.1738

**Published:** 2017-10-02

**Authors:** Muhammad Waqas, Syed Ather Enam, Fauzan A Hashmi, Fatima Mubarak, Fazal Arain

**Affiliations:** 1 Surgery, The Aga Khan University; 2 Radiology, The Aga Khan University; 3 Biomedical Sciences, The Aga Khan University

**Keywords:** glioma, regional sampling, neuronavigation, brightmatter servo, diffusion tensor imaging (dti)

## Abstract

High-grade gliomas possess internal pathological heterogeneity. Selective sampling of different tumor regions can help in the study of this heterogeneity. In this report, we have described the use of a novel navigation and optical system for the selective regional sampling of a high-grade glioma lesion. A 45-year-old gentleman presented to us with complaints of intermittent frontal headaches for past eight months. On examination, he had subtle pyramidal weakness in left upper and lower extremities. Magnetic resonance imaging (MRI) showed a large contrast-enhancing, space-occupying lesion in the right frontal lobe causing perilesional edema and midline shift. We marked four different regions on the preoperative MRI using apparent diffusion coefficient (ADC) mapping and contrast enhancement pattern in four different combinations using presurgical planning software (BrightMatter™ Plan) (Synaptive Medical, Inc., Toronto, Canada). These pre-identified areas were exported into BrightMatter™​​​​​​​ Servo (Synaptive Medical, Toronto, Canada), an integrated robotic video microscope with a neuronavigation system where these areas were selectively sampled and sent for analysis. The BrightMatter™​​​​​​​ Servo not only helped us to the target areas but also helped to identify a safe trajectory, respecting white matter tracts. Histopathology showed a neoplastic lesion composed of mononuclear round cells with the perinuclear halo in a fibrillary stroma with admixed mini-gemistocytes consistent with the diagnosis of a Grade 3 anaplastic astrocytoma. A selective regional sampling of the gliomas can be reliably performed using BrightMatter™​​​​​​​ technologies to study the pathological heterogeneity of these lesions.

## Introduction

Gliomas are heterogeneous in their oncogenic expression and pathological characteristics [1-2]. The differences in tumor regions can be predicted by radiological features of contrast enhancement and apparent diffusion coefficient (ADC) values [3]. Sampling different areas selectively can help us understand the nature of gliomas, culture stem cells, and detect areas of grade change [1].

Diffusion magnetic resonance imaging is a non-invasive tool for mapping the brain based on the diffusion characteristics of water molecules in its various regions [4]. This imaging system shows that the tissue density is inversely correlated with ADC. The variation in ADC has also been shown to reliably predict tumor grade [5] and hence, the nature of these tumors [4]. In 2010, Barajas, et al. studied genetic expressions and ribonucleic acid (RNA) microarrays in different ADC tumor regions and reported variation in their expression [3]. These findings indicate that possible heterogeneity exists in the oncogene expression and tumor stem cells in different areas of the tumor. We have started selective regional sampling of gliomas at our institute to study the molecular and pathological differences that exist within these tumors. To achieve this, we used BrightMatter^™^ Servo (Synaptive Medical, Inc., Toronto, Canada).

BrightMatter^™^ Servo is a novel integrated system comprising of a video microscope (BrightMatter^™^ Drive) that can align to surgical instruments and a neuronavigation system (BrightMatter^™^ Guide) that can display whole brain tractography. The use of BrightMatter^™^ Servo has been demonstrated in both cranial and spinal applications [6-8]. It can, therefore, not only navigate but also focus and visualize the surgical area of interest during surgery. We are reporting the case of a patient who had significant tumor heterogeneity on preoperative magnetic resonance imaging (MRI). We performed selective regional sampling of the glioma using BrightMatter^™^ Servo.

## Case presentation

**Figure 1:**
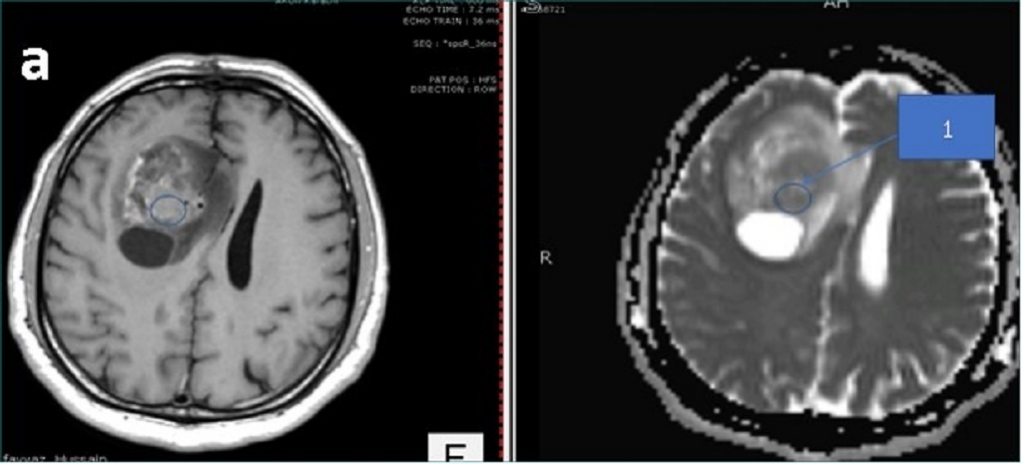
Area 1 The center of the circle has high contrast (left) and low apparent diffusion coefficient map signals (right)

**Figure 2:**
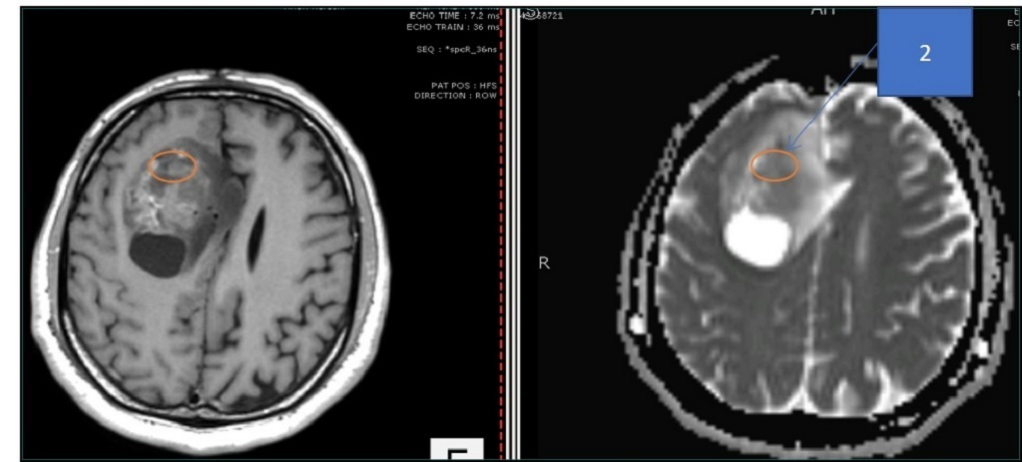
Area 2 The center of the circle has low contrast (left) and low apparent diffusion coefficient map signals (right)

**Figure 3:**
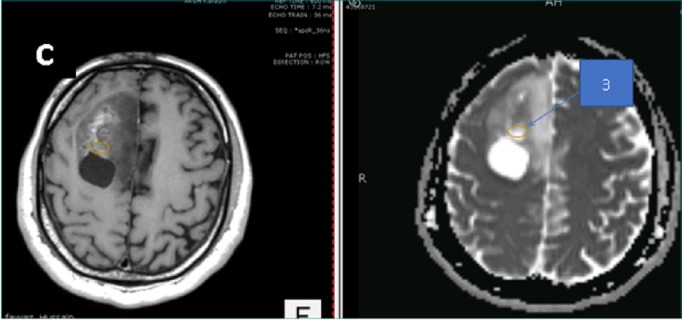
Area 3 The center of the circle has high contrast (left) and high apparent diffusion coefficient map signals (right)

**Figure 4:**
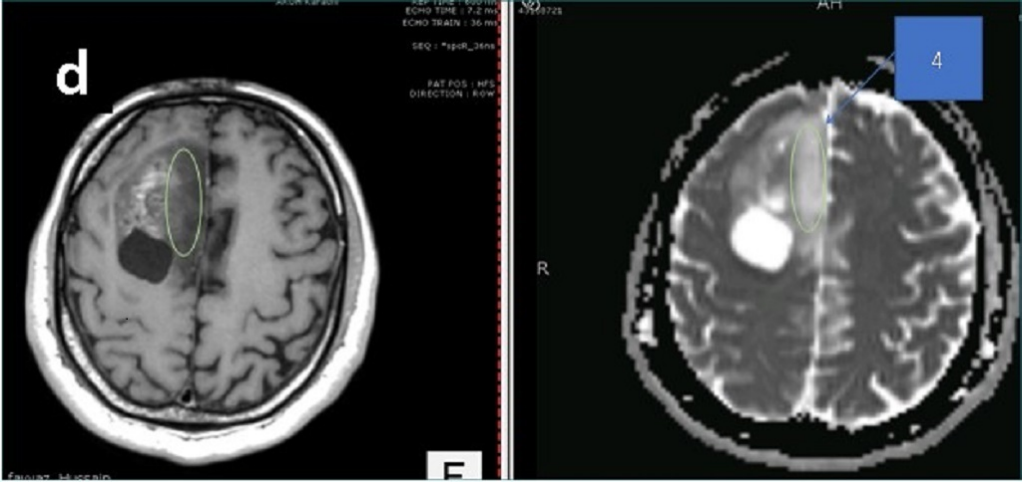
Area 4 The center of the circle has low contrast (left) and high apparent diffusion coefficient map signals (right)

A 45-year-old male patient presented to us with complaints of intermittent frontal headaches associated with nausea and vomiting for the previous eight months. On examination, he was alert and oriented with normal higher mental functions and intact cranial nerves. He had subtle pyramidal weakness in the left upper and lower extremities. MRI of the brain showed a large contrast-enhancing, space-occupying lesion in right frontal lobe causing peri-lesional edema and midline shift. The lesion was also compressing the lateral ventricles. The tractography was generated using the BrightMatter^™^ Plan. This showed the tumor to be displacing rather than infiltrating the right-sided superior longitudinal fasciculus laterally, forceps minor, and genu of corpus callosum posteriorly. The tumor appeared heterogenous on diffusion-weighted images (DWI) and ADC mapping. We marked four different regions on the preoperative MRI using ADC mapping and the contrast enhancement pattern in all four possible combinations. These signal combinations included low ADC and high-contrast (Area 1), low ADC and low-contrast (Area 2), high ADC and high-contrast (Area 3), and high ADC and low-contrast (Area 4). The marked areas are shown in Figures 1-4. This was done using the BrightMatter^™^ Plan where information from different MRI sequences and tractography can be integrated and evaluated to mark different target areas. The patient underwent an awake craniotomy with the help of the BrightMatter^™^ Servo. These pre-identified areas were exported from the BrightMatter^™^ Plan to the BrightMatter^™^ Servo. Using the ADC mapping and tractography, BrightMatter^™^ Servo navigated us to the target areas through a safe trajectory. To minimize the effect of brain shift, samples were taken before debulking of the tumor. Approximately 1 cm^2^ samples were obtained from each of these target areas. The resection was helped by a video microscope. After completion of selective sampling, we continued our resection with the help of the BrightMatter^™^ Servo to achieve a gross total resection.

Postoperatively, the patient remained well; there were no new neurological deficits and the recovery was unremarkable. He was discharged after three days. MRI of the brain done 24 hours postoperatively showed a gross total resection.

Histopathology showed a neoplastic lesion composed of mononuclear round cells with a perinuclear halo in a fibrillary stroma with admixed mini-gemistocytes, consistent with the diagnosis of a Grade 3 anaplastic oligoastrocytoma. Pathological features of different areas are provided in Table 1.

**Table 1:** Different pathological features of selected areas -ve: negative; HPF: high power field

	Area 1	Area 2	Area 3	Area 4
Necrosis	-ve	Moderate	-ve	-ve
Nuclear Pleomorphism	mild	mild	-ve	mild
Mitotic activity	1 in 10 per HPF	-ve	-ve	2 in 10 HPF
Vascularity	Moderate	Mild	-ve	mild

## Discussion

High-grade gliomas are genetically and pathologically heterogeneous lesions [2]. Selective sampling of different regions of the lesion reveals differences in the genetic and pathological characteristics of these tumors [1-2]. Understanding of intratumor heterogeneity is critical to future breakthroughs in medical and surgical therapies [1-2]. This intratumor heterogeneity is, however, understudied [1]. One of the reasons has been the difficulty in navigating targets and increased risk of injury to normal structures. Neuronavigation facilitates the process of regional sampling of the tumors. It helps in targeting different areas. In most cases, however, visual aid, i.e., microscope, is not integrated with the neuronavigation. This makes accurate sampling difficult. BrightMatter™ Servo has a video microscope on a robotic arm (BrightMatter™ Drive), which is integrated with neuronavigation (BrightMatter™ Guide). Both the navigation image and operative view are displayed together on the same screen. We selected different regions of interest on the BrightMatter™ Plan, exported the plan to BrightMatter™ Servo, and the robotic arm guided us to those areas. BrightMatter™ Servo was especially useful for selective sampling more than the conventional navigation as we could mark the targets on the navigation images and it informed us about the anatomical relations of each of the target areas for improved safety. However, we do not have a direct comparison with other methods of selective sampling.

The BrightMatter™ Plan can be used in the analysis of tractography, preoperative MRI images, identification of targets, and choosing safe trajectories to each of the targets. The plan thus made can be exported to the BrightMatter™ Servo where the information can help navigate (BrightMatter™ Guide) and perform sampling (BrightMatter™ Drive). The risk of injury to normal cortex and white matter while attempting selective sampling of different areas can be minimized using this integrated system.

Use of a video microscope is becoming popular among neurosurgeons [6-8]. However, this is the first report describing the use of video microscope for a regional sampling of glial neoplasms. With increasing use of a video microscope, it has been pointed out that the system lacks the adequate stereoscopy. This limitation of the optical system, however, does not hamper the regional sampling as both the superficial and deep lesions can be accessed with equal ease using neuronavigation. We believe that this novel system can really help future studies on tumor heterogeneity.

## Conclusions

The selective regional sampling of the gliomas can be reliably performed using BrightMatter^™^ Servo to study the pathological and genetic heterogeneity of these lesions. The technique can potentially minimize the errors of sampling while performing a tumor biopsy.  
